# Machine learning and health need better values

**DOI:** 10.1038/s41746-022-00595-9

**Published:** 2022-04-22

**Authors:** Marzyeh Ghassemi, Shakir Mohamed

**Affiliations:** 1 Department of Electrical Engineering and Computer Science, Massachusetts Institute of Technology, Cambridge, MA 02139 USA; 2 Institute for Medical Engineering & Science, Massachusetts Institute of Technology, Cambridge, MA 02139 USA; 3 CIFAR AI Chair, Vector Institute, Toronto, Ontario M5G 1M1 Canada; 4 DeepMind, 5 New Street Square, London, EC4A 3TW UK

**Keywords:** Culture, Health care, Medical research

## Abstract

Health care is a human process that generates data from human lives, as well as the care they receive. Machine learning has worked in health to bring new technology into this sociotechnical environment, using data to support a vision of healthier living for everyone. Interdisciplinary fields of research like machine learning for health bring different values and judgements together, requiring that those value choices be deliberate and measured. More than just abstract ideas, our values are the basis upon which we choose our research topics, set up research collaborations, execute our research methodologies, make assessments of scientific and technical correctness, proceed to product development, and finally operationalize deployments and describe policy. For machine learning to achieve its aims of supporting healthier living while minimizing harm, we believe that a deeper introspection of our field’s values and contentions is overdue. In this perspective, we highlight notable areas in need of attention within the field. We believe deliberate and informed introspection will lead our community to renewed opportunities for understanding disease, new partnerships with clinicians and patients, and allow us to better support people and communities to live healthier, dignified lives.

## Introduction

Many of the key values of health care—e.g., respect for persons, beneficence, and non-malfeasance (do no harm)—are widely known, having been established over the last century following significant historical events, including the Nuremberg Code, the Declaration at Helsinki, and the Belmont report, and others. The key values of machine learning (ML), among others, could be said to be those of openness, reproducibility and scalability. As a field, ideally ML for health productively merges these constitutive values. Yet, because ML and health exist in a wider social context, other values—of efficiency and cost-effectiveness for providers, care value for patients, and the commercial interests of ML solutions developers—are also infused into the field’s research and products.

In this perspective we explore the tensions that make it difficult to implement key values of ML and healthcare as both fields expand and intersect to incorporate the key values of the other. We present a summary of our sections in Table 1, and note the tensions highlighted in each. For instance, while there is clear scientific value to openness, there are biased legacies inherited from colonial health datasets that are collected under medical inequity in the past and present. We also highlight examples of different approaches to improved values, for instance by using ML to maintain and enhance human dignity, and conclude that new ideas are needed to pave the way for research that is more sensitive to risks and harms, improves accountability to patients and the public, and enhances our ability to conduct new types of health care research.

**Table 1:** Summarising the values, tensions and descriptions used.

Section	Tensions	Description
Values of Openness	Fast-paced iteration vs deployed validation Knowledge vs privacy	Rapid scientific iteration is in tension with the need to carefully validate deployed model performance. Widespread scientific knowledge generation is in tension with the need to preserve individual privacy.
Biased Legacies in Datasets	Use of existing data vs replicating colonial medicine	Using the existing data we have is efficient, but can extend the biased legacies in datasets stemming from “colonial medicine”.
Improving Medical Evaluation with Machine Learning	Commercialization vs. open research Addressing vs extending human mistakes	Commercialization of medical learning systems is in tension with open release of data and knowledge. This is furth complicated by patient consent. There are approaches for redressing existing disparities in treatment rather than extending them.
Maintaining and Enhancing Human Dignity	Efficient re-use of data vs. scientific representation	Co-opting existing data or systems for use in research that a group does not agree to is efficient, but may disregard the trust and dignity needed towards human subjects.

A partial summary of initial topics that are central to machine learning for health.

## Values of openness

The ML community as a whole has generally embraced the concept of openness as a key value: it is common for code to be “open-sourced”, datasets to be publicly released, and paper preprints to be posted on freely available internet archival services. This is often all completed before a paper is published. Sometimes code, data, and paper are all posted online when a submission is made, despite the potential for the paper to be rejected. In this setting of radical openness, the value of getting an idea out first is paramount. These practices have undoubtedly quickened the pace of ML research: any researcher expects to be able to reproduce results on a paper preprint within a day if a pre-trained model is available, and perhaps a week if any local training is required. Potential drawbacks of this increased research pace include the potential for strong empirical results in a specific, targeted setting to be pushed into the community before there is the opportunity for more depth and introspection about their meaning. This is an important caveat to our cited ML values—models, algorithms and systems are often evaluated within large data ecosystems, but strong empirical results are not necessarily replicable^1^. Given this backdrop, ML researchers working in health have struggled to determine the right cadence of research openness and, as a result, pace of innovation. While researchers should not blindly apply the same levels of openness to enable fast-paced research, health has notably been lagging in openness metrics^2^ driven in part by relatively few available data sources with the same openness standards^3^.

Radical openness raises concerns in health settings due to a fundamental tension with deployed validation. In an era where anyone with an internet connection and a little tenacity can download, build, and deploy state-of-the-art algorithms, a model *intended* for more efficient allocation of health resources can disenfranchise the communities it is ostensibly meant to serve^4^ once deployed. This would result in algorithms that are meant to provide improved diagnostic accuracy instead perpetuating bias and eroding public trust. The importance of iterating quickly in machine learning must therefore be considered in proportion to the high level of evidence required for clinical deployments. In other words, more time-consuming study designs may be needed in many medical settings in order to best integrate new findings into practice. In high-stakes settings like aviation or space exploration, the practicality of implementing technical support tools and the implications of these tools on practice is rigorously studied. In machine learning, such implementation science questions are often overlooked by researchers and left for later “implementers” to consider, despite the reality that positive implications for care depend on a tool’s ability to trigger actual human workflows and change human behaviours.

Openness does not only impact efficiency, but can help multiple teams work on a problem collectively, and advance knowledge. While privacy is a commonly-cited concern with health data^5–7^, patient privacy can be also used by data-holding institutions as a barrier for data access, while these same entities sell data or use it internally for their own research advancement. Purely technical solutions like federated learning or differential privacy are poor fits because they do not address the underlying barriers to access. For instance, if Hospital A holds patient data that it routinely sells to data aggregators at a high cost, or provides only to its own affiliated researchers in an effort to increase the scientific value of its institutional contributions, offering a federated solution is not helpful. Issues of incentives, profits, and intellectual property cannot be technically addressed if the fundamental tension is a sociotechnical one of value conflict. Instead, data holders must be encouraged by regulatory bodies to provide data under relevant and meaningful legal protections to researchers. While individual countries may vary in how this line is drawn, we would encourage regulatory bodies to make clear rules about the ways in which data can and should be made publicly available for audit and advancement of scientific contribution by public and private bodies.

## Biased legacies in datasets

ML research has benefited from increasingly large labelled datasets; however, researchers must carefully consider which datasets are used for training. In this setting, ML researchers must consider ethics and the valuation of human dignity in all stages of the development pipeline—problem selection, data collection, outcome definition, algorithm development, and post-deployment^8^. The potential for naive assumptions to cause actual harm can be addressed by expanding approaches to inclusion, careful auditing of development, and more sophisticated performance analyses.

Learning from data collected in the past can generally be dangerous when applied to biased data without careful thought^9^, or used by those with an intention to stigmatize a subgroup^10^. In health care specifically, learning from historical data also comes with its own baggage, and this baggage may mean that radical openness brings unwanted disadvantages. Health research is not free from the problems of biased data, or subgroup stigmatization. Classic health datasets are biased by cohorts that were predominantly white men of European descent^11^, and by the societal biases that minorities and minoritized patients are subjected to^12^. Risk scores in clinical areas ranging from cardiology to obstetrics that use racial identity as part of their estimation have important equity concerns for both over- and under-treatment^13^. These issues are not limited to past interactions: individual doctors can have strong preferences about patients^14^ or treatments^15^, and standard medical practices are often disaffirmed by later research^16^.

While these are concerns that are not specific to ML, the automation potential of high-capacity ML models in these settings demands that we ask questions about what sort of practices we will be automating. Researchers in health care have inherited a centuries-long legacy of data steeped in racism and sexism that perpetuates into the present. Any work that seeks to improve technology in this space must grapple with the historical context of colonialism and colonial medicine^17–20^. The first genocide of the 20th century in Namibia involved developing new methods of medical sterilisation, where science was used to justify racial ideology above the value of equality^21^. The case of the US Department of Health studying syphilis in Tuskegee in which African-American men were left with untreated syphilis is one of the most widely-known cases of medical harm, and was driven by the value of gathering more data above that of human dignity^22^. More recently, there remain ongoing questions of illegal blood exports during the 2014–2016 West Africa Ebola epidemic, privileging the value of data over consent^23^. Even as recently as the COVID-19 pandemic, scientists suggested that vaccines for COVID-19 should first be tested in Africa, privileging the value of western, developed countries’ safety, over global health equity^24^. Biases in health pipelines are directly relevant to researchers who must work to understand the hidden perils of datasets they might have access to^25–31^.

As the scholar of race and technology, Ruha Benjamin, recently noted^32^: if deep learning models are simulating a mind built on large amounts of data from bodies and behaviours, whose minds and bodies and behaviours are being simulated?

## Improving medical evaluation with machine learning

Automation of medical evaluation can encode societal biases and inequities without proper consideration, and many patients worry that their autonomy and choice could be further compromised if ML systems are used in healthcare settings^33^. Importantly, in large health care systems such as those in the US or the UK, there are both financial and commercial interests in play that may change research and researchers.

Commercialization versus the contribution of knowledge through scientific discourse is an issue that is particularly relevant for machine learning in health, due to balancing concerns in improving the experience of care, improving the health of populations, and reducing per capita costs of health care^34^. The best possible value-for-money care is often prioritized over other values such as racial equity or patient trust, and learning models based on such data will lead to similar judgements from an ML system. Commercialization of products can also come into conflict with the idea of open ethical research, if such efforts are not well-motivated. In the absence of regulation, we believe that standards should be agreed upon and adopted by the research community to encourage innovation and scientific translation. Underlying the tension of data commercialization is the further issue of consent, especially in the face of commercial interests. The use of opt-in research is one approach that has been used to establish large, diverse samples of human data, with efforts such as the US National Institute of Health’s All of Us Research Program and the UK Biobank^35^ having invested great effort to recruit and retain such consented samples. While All of Us and Biobank included explicit consents for use of patient biological samples, much electronic health records research more generally, is not done with explicit consent.

We can see the effect of different approaches to values that transcend the status quo of biased health in some recent work. Obermeyer et al.^4^ report on a ML algorithm that was developed to identify high-need patients eligible for a care management programme that allocated additional medical resources. Instead of relying on the patient’s physiology to determine who was at high risk of worsening health, designers relied on data from insurance claims. By using spending as a proxy for need, the algorithm effectively discriminated against Black patients, and both exposed and perpetuated racialized disparities in the US health care system. A very different outcome is reached when the needs of patients, especially those of underserved patients, is taken into account. Peirson et al.^36^ show that intentionally using data from underserved populations closed disparities in pain scores seen across dimensions of race, lower-income, or educational attainment. In these two approaches, we see clearly that there are approaches and alternatives for redressing disparities in treatment that are already available, and that meaningfully avoiding harm is fully possible.

What these examples illustrate is that inequities in health care have historically been delivered, in part, due to the inherent noisiness of human judgements^37^. The harms that can arise from medical ML, like performance differences in chest X-ray diagnostics^10,20^, are not an aberration or unintended consequence. Applying ML, in and of itself, can continue or add to the known forms of injustice and harm in health, now in technological and automated forms. On the other hand, researchers have already demonstrated the ability to harness the gap between models doing as doctors do—rather than as we say doctors do—to identify harmful effects of current medical care.

## Maintaining and enhancing human dignity

Machine learning researchers working in health have a crucial role in maintaining and enhancing human dignity throughout the research process. Beyond the inherent issues in biased data collection that must instead respect human dignity over data value, there is a further need to examine the context in which collected data is used. In health care, there are moments where you must share information, e.g, with a doctor or nurse. However, moving beyond that context without examination is ethically challenging. For instance, we may accept that researchers will use our data to generate public knowledge, but not want any private company to have access to our medical data, even if it is de-identified, because we don’t want to be targeted for ads related to our health conditions.

Researchers may also misuse data in contexts that put the consented population at risk or remove their dignity in favour of curiosity. In the 1980s researchers collected genomic data from the Havasupai tribe on the basis of identifying potential genetic links to diabetes risk, and data from the Nuu-chah-nulth in British Columbia for genomic research on rheumatoid arthritis. In both cases, no useful link was found, and genetic data were then used to study unrelated topics such as schizophrenia, inbreeding, migration, HIV status, and drug abuse (https://www.vice.com/en/article/8xp33a/the-nih-is-bypassing-tribal-sovereignty-to-harvest-genetic-data-from-native-americans). The data was used outside of the context it was intended for, breaking the trust and right to dignity of the people who provided it. Beyond research, models can inform policy directives or be used for health surveillance, needing researchers to consider the implications of removing subject choice and privacy, often from those who are most vulnerable and with the least power. These examples show that even if researchers intend to perform research that is valuable to a community and appropriately collect data, defining the ongoing context for data use post-collection is an open problem with no clearly optimal way to consistently obtain meaningful consent.

## Conclusion

It is worth reflecting on why or how values are codified in a community, because this is often dictated by who in the community has power. For instance, if peer review is done by junior researchers then more recent high-risk/high-reward topics and techniques may be scored better; if research on clinical problems from specific communities are disproportionately funded then clinicians may specialize in those fields to the exclusion of others; if performance of technology is regulated by averages then companies may neglect rigorous guarantees in minority and minoritized populations. In these cases, explicit regulatory requirements by relevant bodies is a potential solution, for instance, the FDA may require transparency around subgroup stratified model performance to mitigate model bias in pre- and post-deployment settings.

Implementing justice and addressing the inheritance of colonialism will continue to be among the most challenging forms of change, but is necessary. Researchers must more deeply interrogate the human judgements and social systems that give rise to the data and treatment policies used as labels and targets. This is where ML can advance equity in medicine, by bringing the open-science principle into use in ways that reveal patterns of incidence and treatment that may be hidden. Neither ML nor medicine alone can navigate the complexities of opportunity and harm that arise from this sociotechnical system. Minority or minoritized performance differences found in health datasets should not be taken as biological fate, but rather studied as the cumulative effect of the experience of being in unequal societies. If ML has learnt associations that we are uncomfortable with, this provides us with an opportunity to raise the bar for accountability in medicine. For instance, if it makes us uncomfortable that a model is making poorer judgement calls on Black women, we can audit clinical performance to alert or train doctors to be aware that they are part of creating those data-driven patterns at the local or regional board level.

If we do not deal with the implications of how humans treat each other now, then it should be of little surprise when data that demonstrates poor treatment becomes automated, exported, and moved into production. Important issues around patient consent, data commercialisation, research openness, and many others, could be explored as foundational topics through research panels led by international teams of researchers. This approach has been used in other settings to create standards for quality or performance such as the SPIRIT-AI and CONSORT-AI^38,39^ statements, and could be extended further to guide researchers. Identifying where dignity should be injected into both ML and health care processes will improve not only models and research, but the lives of people who are interacting with and affected by them. There are clear tensions between knowledge and openness versus privacy, objectification, and consent to navigate here. And with deeper interrogation, participation, and a focus on dignity, we put the project of ML for health onto a clearer path towards supporting healthy living for all.
